# Material perception across different media—comparing perceived attributes in oil paintings and engravings

**DOI:** 10.1177/20416695241261140

**Published:** 2024-08-03

**Authors:** Yuguang Zhao, Jeroen Stumpel, Huib de Ridder, Maarten W. A. Wijntjes

**Affiliations:** Perceptual Intelligence lab, 578936 Faculty of Industrial Design Engineering, Delft University of Technology, The Netherlands; Department of History and Art History, 8125 Utrecht University, The Netherlands; Perceptual Intelligence lab, 578936 Faculty of Industrial Design Engineering, Delft University of Technology, The Netherlands

**Keywords:** three-dimensional (3D) perception, colour, surfaces/materials, texture, art and perception, pictorial media

## Abstract

We investigated the influence of the medium on the perception of depicted objects and materials. Oil paintings and their reproductions in engravings were chosen because they are vastly distinctive media while having completely identical content. A total of 15 pairs were collected, consisting of 88 fragments depicting different materials, including fabric, skin, wood and metal. Besides the original condition, we created three manipulations to understand the effect of colour (a greyscale version) and contrast (equalised histograms towards both painting and engraving). We performed rating experiments on five attributes: three-dimensionality, glossiness, convincingness, smoothness and softness. An average of 25 participants finished each of the 20 online experimental sessions (five attributes X four conditions). Besides clear correlations between the two media, the differences mainly show in their means (different levels of perceived attributes) and standard deviations (perceived range). In most sessions, paintings depict a wider range than engravings. In addition, it was the histogram equalisation (global contrast) that made the most impact on perceived attributes, rather than colour removal. This suggests that engravers compensated for the lack of colour by exploiting the possibilities of local contrast.

Around the time that in Italy linear perspective was discovered (Alberti, 1966), a material rendering innovation was taking place in Northern Europe mediated by the invention of oil paint. Although he may not have been the inventor, van Eyck was certainly the artist who discovered the huge potential of oil paint for the convincing rendering of materials. The deeper colours and the slow drying, which enabled smooth transition and easy alteration, offered artists possibilities that did not exist for tempera paint (Bol, 2023). While the invention of linear perspective was related to the mathematics of projection, the material rendering revolution was related to a specific medium: that of oil paint. The perceptual influence of media is a relatively understudied topic and we made that the topic of the current paper. However, instead of comparing oil and tempera, we choose two media that are more distant from each other: oil paintings and engravings. In the context of this research, the term “engraving” specifically refers to monochrome engravings, and explicitly excludes any painted or colour-printed engravings.

The artistic handling of a medium is related to the topic of style. Within a certain medium, such as oil paint, there are obviously many different styles as art history has shown. We previously found a relation between differently depicted apples and their material properties (Zhao et al., 2023). van Zuijlen et al. (2020) took a different approach by collecting a large variety of annotated material segments from historical oil paintings. They collected material attribute ratings for 15 different material classes and compared them to a study of a similar nature that used photographs (Fleming et al., 2013). Interestingly, the material “signatures” (perceptual characterisation of 10 material attribute ratings) are very similar between paintings and photographs, suggesting material perception might be independent of the medium. Instead of comparing paintings and photographs, in a more controlled fashion Delanoy et al. (2021) compared realistic material computer renderings with their painting replicas by an artist. They reached the conclusion that material properties in paintings and renderings were perceived very similarly and were linked to the same image features. While these studies suggest that material perception might be independent of media, Bousseau et al. (2013) found differences between realistic renderings and painterly renderings of the same scenes. Their results showed that in painterly renderings, the range of distinguishable gloss levels reduces under increased brush size of opaque strokes, use of semitransparent strokes, or when textures of brush strokes and varnish were introduced.

The different conclusions from previous studies left the question unanswered whether the medium has an influence on material perception. In the current study, we wanted to answer this question using the same variety of depictions as van Zuijlen et al. (2020) while comparing different media. At the same time, we also desired that the subject matter could be kept constant as was achieved by Bousseau et al. (2013) and Delanoy et al. (2021). The requirement of identical subject matter was difficult to meet, since artists generally compose original pictures which do not share a perfect subject matter resemblance.

We found a solution by comparing paintings and their reproductions in print media, particularly engravings. Engraving has been a form of art on its own, but also as a method to reproduce paintings from the 17th century onwards, before various printing techniques that made direct use of photographic images, from rotogravure, to off set and beyond. The identical pictorial content in oil paintings and their engraved reproductions provides a perfect opportunity to compare the portrayal of materials such as fabric and skin across the two media without the confounding factor of different subject matter. Maintaining constant subject matter would be much more difficult, if not impossible, if we were to compare oil and tempera paint. Furthermore, the two media are drastically different, which makes it a critical case study for the influence of media on perception.

Seemingly originating from goldsmithing, engraving emerged in the late 15th century in Germany and Italy. As an intaglio process, engravings are created with a burin, a wedge-shaped metal tool, to carve into the base plate usually made of copper. The plate, consisting of grooves created by burin, could hold ink. Ink would then transfer onto a damp sheet of paper under high pressure to complete a print. The early German master Martin Schongauer raised engraving from a minor craft to a major art form with compelling works, followed by Albrecht Dürer, and many other masters (Thompson, 2000). The process of engraving differs from etching. In etching, the metal plate is covered by a layer of wax or soft varnish. The artist can draw effortlessly by removing parts of this layer with a needle, upon which a chemical process with acid creates the grooves. However, in engraving the grooves are made directly by the handling of the burin which requires great skill and craftsmanship, based on years of training.

Engraving is a challenging medium not only because of the difficulty in craftsmanship, but also because it is a medium restricted by monochromatic lines and dots. Oil paintings have coloured fluid brush strokes and can easily achieve smooth colour transitions and colour contrast. Engravings, on the other hand, are categorically different, with only “black” and “white” (the colour of the ink and the paper). Luminance contrast is achieved by the distribution of lines. Within these boundary conditions, engravers were still able to create form, texture, shading and highlights. Engravers had their own idiosyncratic approach to creating engraving lines, some preferred to use lines that followed the contours, and some preferred cross-hatching to create shading and three-dimensional (3D) volume (Thompson, 2000).

To quantify the perceptual differences between paintings and engravings, we focused on measuring five perceptual attributes of various depicted objects. We investigated the depiction of materials by letting observers rate the smoothness, glossiness and softness. Furthermore, we let observers rate three-dimensionality to assess the depiction of shape. In addition to investigating the formal elements of material and shape, we were also interested in the overall quality of the depictions of objects. Therefore, we asked observers to rate the “convincingness.” We will shortly elaborate on these five attributes.

Since many old masters in both painting and engraving pursued realistic and convincing depictions, we compared the convincingness of these two media. As an overall judgement, convincingness (or realism in different terms) has been widely studied in the field of visual perception (Berlyne Ogilvie, 1974; O’Hare Gordon, 1977; Chatterjee et al., 2010; Di Cicco et al., 2018; Di Cicco, 2022) and is often considered an important perceptual measurement. It should be noted that convincingness seems to play a role both in historical pictorial revolutions such as the invention of linear perspective and oil paint as discussed above, but also in contemporary pictorial revolutions. The recent success of artificial intelligence-mediated synthetic image algorithms such as Midjourney is largely attributable to their impressive convincingness (Göring et al., 2023).

Gloss is the most widely studied attribute that is important for material perception (Marlow Anderson, 2013; Pellacini et al., 2000), including real and photographed objects (van Assen et al., 2016; Zhang et al., 2019), computer rendered images (Wendt et al., 2008), and also for paintings (Di Cicco et al., 2019; Bousseau et al., 2013; Delanoy et al., 2021). Previous studies concluded that gloss perception is mostly determined by contrast, sharpness and coverage of the highlights (Marlow et al., 2012; Di Cicco et al., 2019). Contrast, which is manifested distinctively in oil paintings and engravings, plays a pivotal role as one of the key features and predictors of gloss perception (Di Cicco et al., 2019).

Smoothness plays an important role in perceived realism (Rademacher et al., 2001). Sometimes it has been measured as its opposite, roughness (Delanoy et al., 2021; Di Cicco et al., 2021; Zhang et al., 2019). What is furthermore interesting about smoothness is that it can refer not only to the smoothness of the depicted object (the motif), but also to the depiction (the medium). This could theoretically also be the case for gloss, but the glossiness of the medium (e.g. caused by the varnish) is often made invisible by way of visual documentation: a glossy reflection in a photo copy of a painting is rather undesirable. However, the roughness of brushstrokes or hatching is difficult to ignore. Interestingly, Zhao et al. (2023) found a potential transfer of smoothness between medium (smooth brushstroke) and motif (depicted apples) in a study on style perception. In the current study, we were interested in whether the visible engraving lines in the medium (see Figure 6) may influence the perceived smoothness of the depicted materials.

The third material attribute that we decided to investigate is softness. It is particularly related to materials such as fabric and skin, which make up the larger part of our stimulus set. Previously, it has been found that softness is not correlated to roughness in a study on depicted fabric perception (Di Cicco et al., 2021). Furthermore, softness could be seen as a more mechanical property as opposed to the optical property of gloss. Hence, softness complements the other two material attributes rather well.

A related attribute, though not a material attribute but rather a shape attribute, is three-dimensionality. There is a strong perceptual connection between gloss and 3D shape (Fleming et al., 2004; Todd Mingolla, 1983; Norman et al., 2004). Contrast is also used as an effective depth cue for 3D shape perception (O’Shea et al., 1994). Since engraving has different approaches than oil painting to achieve 3D rendering, we will investigate the performance of the medium in expressing three-dimensionality.

There are a number of a priori differences between paintings and engravings that could lead to perceptual differences. Colour and contrast are the most prominent differences. Being denied access to colours, engravers likely compensated by deploying all available efforts towards the luminance channel. While we empirically investigated the “original” (albeit digitised) pictures, we additionally included image manipulations to better understand the respective roles of colour and contrast.

The first image manipulation served to understand the role of colour and consisted of taking grey scale versions of both stimuli. This was established by converting the colours into luminance values. To understand the role of luminance contrast we equalised the respective luminance histograms. However, because the luminance histogram of an engraving theoretically consists of two single peaks at the white of the paper and the black of the ink, we first blurred the engraving such that hatchings became smooth gradients. To counterbalance the blurring manipulations on the engravings, we applied the same procedure to the paintings. In sum, we added two manipulation conditions to the original condition: greyscale and equalised luminance histogram. As the latter condition can be applied both from the engraving to the painting and vice versa, this condition consisted of two versions. Thus, a total of four conditions (original, greyscale and two histogram equalisations) were measured in the following experiment.

## Method

### Stimuli

**Figure 1.**
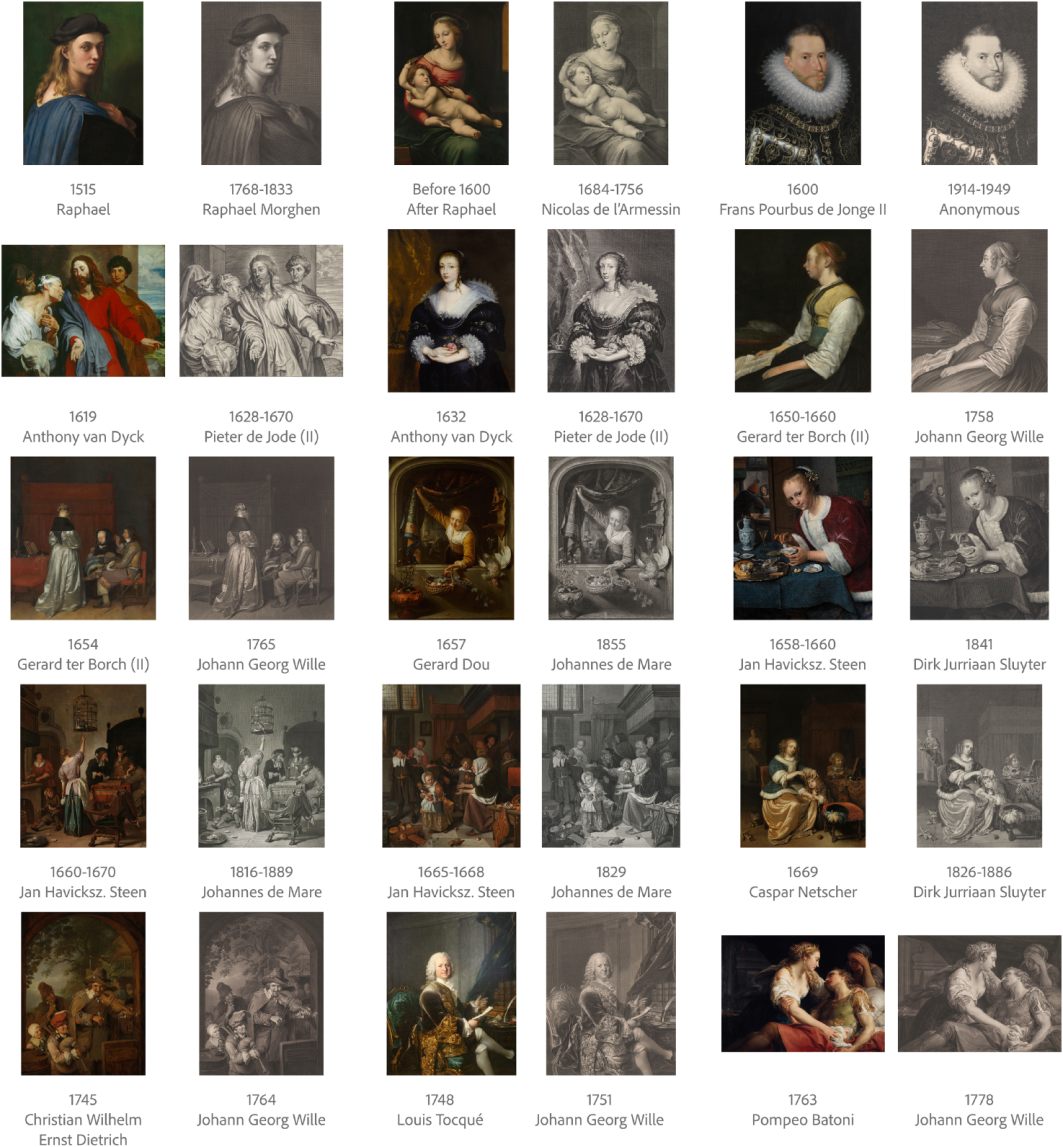
An overview of 15 pairs of stimuli, sorted by creation year of oil paintings. In some cases, where there is no precise creation year information available, we presented the estimated range of creation year or the lifespan of the artist.

We collected 15 pairs of digital copies of colour oil paintings and their engraving reproductions. Identical content gave us the opportunity to take medium as a controlled variable and minimise the influence of content, or “subject matter.” Most oil paintings are portraits or scenes of daily life to ensure the diversity of materials. Both original oil paintings and their engraving reproductions covered a wide range of creation years. The creation year of the original oil paintings varied from the 16th to 18th century, while the creation year of engravings ranged from the 17th to 19th century. Figure 1 shows an overview of all stimuli.

Before further processing, we first endeavoured to align all the pairs and crop them into the same framing. Since engravings are not photo copies, their framing and aspect ratio can differ slightly from the original oil paintings. Better aligned content can further reduce the influence of subject matter. A few images were mirrored for the alignment. Besides, some engravings have text below the figures, which is different from oil paintings. Removing text reduced the possibility for participants to easily infer the media. All the aligned and cropped high-resolution images were then rescaled in Adobe Photoshop (Adobe Inc., 2021). Since they have different aspect ratios, we set the longer edge to be 1500 pixels.

Then we created two manipulations to understand the effect of colour and contrast. Firstly, we removed chromatic information by creating a greyscale version. The conversion was performed in Mathematica (Wolfram Research Inc., 2020), the formula from standard red–green–blue (sRGB) to greyscale is Gryscale=0.299R+0.587G+0.114B . Secondly, we removed the difference in global luminance contrast by equalising histograms (towards both painting and engraving, hence two versions). The histogram matching was performed with the “HistogramTransform” function in Mathematica (Wolfram Research Inc., 2020). Before the histogram equalisation, we removed high-frequency information by blurring the images. This was necessary as a “sharp” engraving is essentially a bitmap: black when there is a line, white in the background, without intermediate greyscale values which only emerge when viewed from a distance, that is, blurred. The Gaussian blur radius for each stimulus was determined per picture individually so that engraving lines became invisible. It should be noted that the purpose of the blurring was to facilitate histogram matching, and not a purpose on its own, hence we only have two image manipulation conditions: greyscale and histogram equalisation. That we end with four experimental conditions (including the “original” condition) is due to histogram equalised images having two versions with either the painting or the engraving functioning as a source.

After the manipulations, all stimulus images were converted in Photoshop (Adobe Inc., 2021) to PNG format, and embedded with an sRGB International Color Consortium colour profile, for browsers to display colours properly (Ashe, 2014).

Lastly, from each picture pair we selected multiple objects, including fabric, skin, lace, wood, metal and ceramic, marked with a red outline in the experiment interface (see Figure 3). In total, we selected 88 objects from these 15 pairs. Table 1 shows the numbers of selections in detail. A preview of all 88 selections can be found in the supplementary material.

**Table 1.** Number of selections for each material category.

	3D/gloss/convincingness/smoothness	Softness
Fabric	54	54
Skin	18	18
Lace	4	4
Fur	2	2
Metal	3	na
Wood	6	na
Ceramic	1	na

### Experimental Design

**Figure 2.**
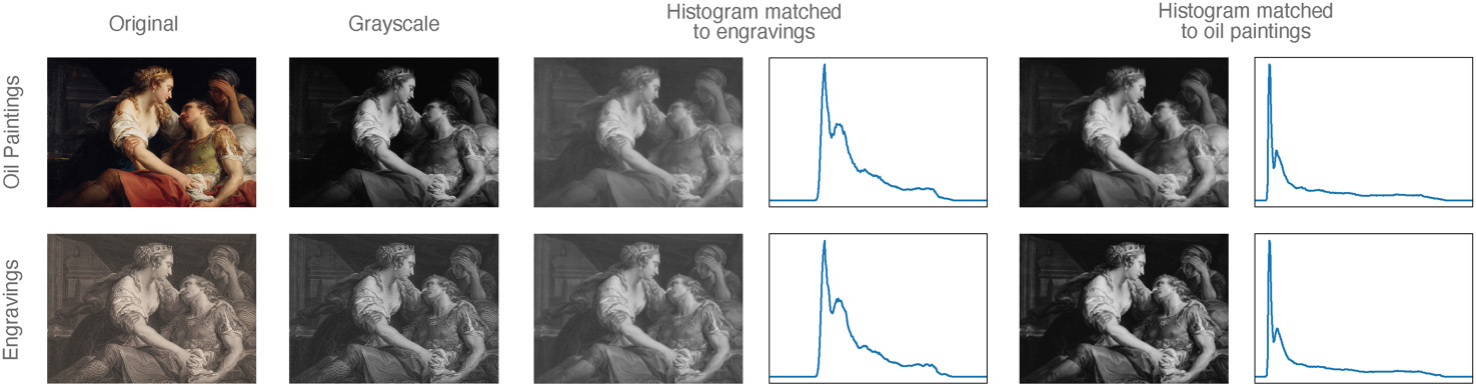
Four conditions: original, greyscale, histogram of painting matched to that of engraving (hmp), histogram of engraving matched to that of painting (hme). For hmp and hme conditions, we first applied the same Gaussian blur to both engravings and paintings before histogram matching so that the engravings have smooth histograms and no visible engraving lines. After histogram matching, they have the same overall luminance distribution. Note that blurred oil paintings usually have higher contrast than blurred engravings. The oil painting: Pompeo Batoni, *La mort de Marc Antoine*, 1763. Downloaded from Wikipedia. The engraving: Johann Georg Wille, *La Mort de Marc Antoine*, 1778. Downloaded from the online repository of the Rijksmuseum, Amsterdam. Both images were cropped to the same framing.

The study consisted of 20 online experimental sessions. In each session, a unique group of participants judged one of five attributes for the two media (oil paintings and engravings) in one of four conditions: original (ori), greyscale (bw), histogram of painting matched to that of engraving (hmp), histogram of engraving matched to that of painting (hme) (see Figure 2). Per attribute, this resulted in a 2×4 mixed design, with medium as a within-subject and condition (manipulations) as a between-subject variable. The five attributes to be judged were: three-dimensionality, glossiness, smoothness, softness and convincingness. Each attribute scale was defined by two contrasting terms, listed in Table 2 as left and right labels at either end of the continuous rating scale. No additional information was provided about the attributes to be assessed.

**Table 2.** Keywords of rating scales for attributes rating.

Attributes	Left label	Right label
Three-dimensionality	Flat	Three-dimensional
Glossiness	Matte	Glossy
Smoothness	Rough	Fine
Softness	Hard	Soft
Convincingness	Unrealistic	Realistic

All attributes have 88 material selections in total except softness which has 78, since metal, wood or ceramic is not relevant for softness. As a result, three-dimensionality, glossiness, smoothness and convincingness had (88 times two) 176 trials and softness had (78 times two) 156 trials for each session.

### Participants

Six hundred unique participants were recruited for our experiment, 30 participants for each experimental session. However, we lost some responses due to server issues which resulted in an average of 25 participants for each session. All participants were recruited from Prolific (www.prolific.co) from all available countries. The experiment was conducted in agreement with the Declaration of Helsinki and approved by the Human Research Ethics Committee of the Delft University of Technology. All data were collected anonymously.

### Procedure

**Figure 3.**
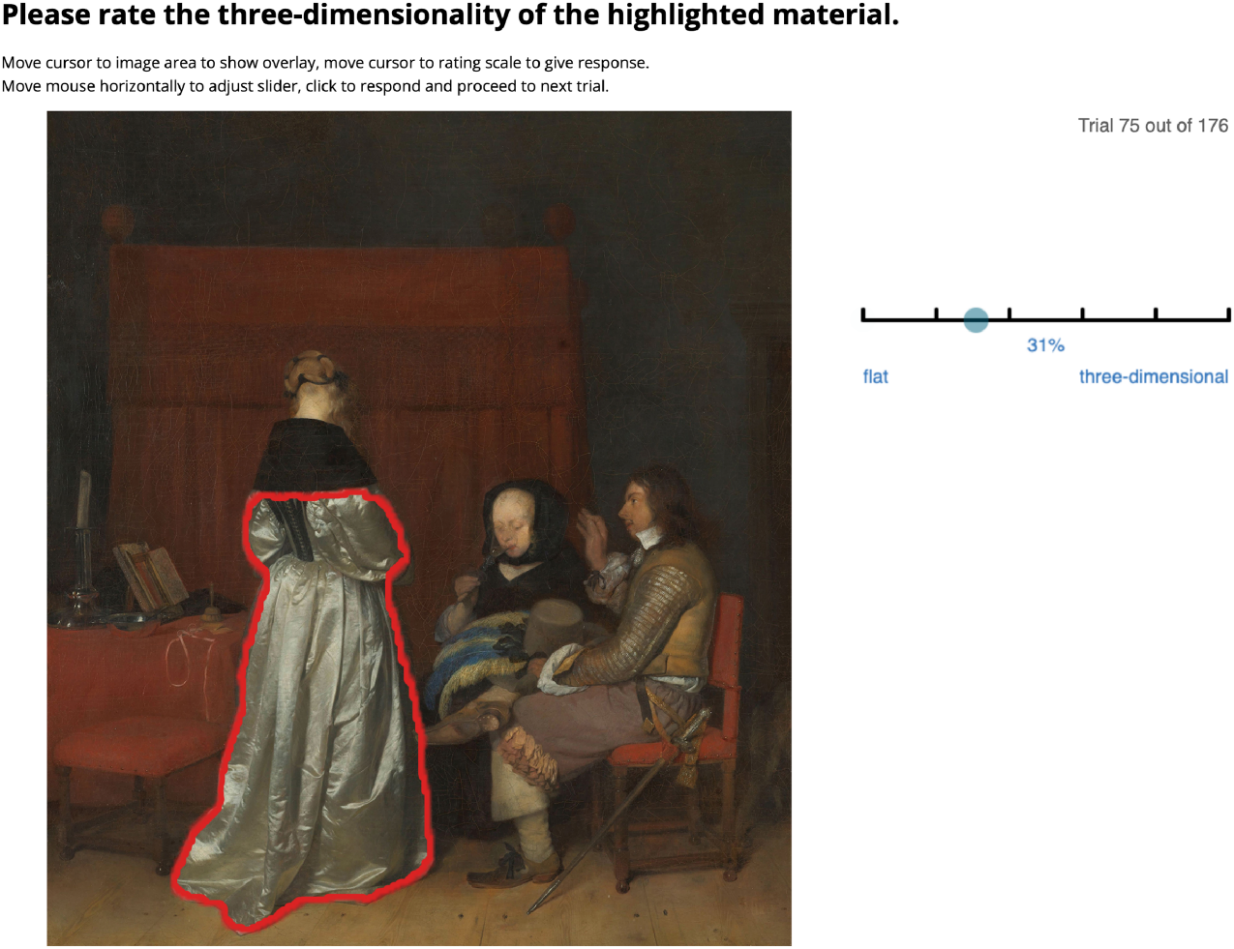
Experiment interface of original condition regarding three-dimensionality. Each time a new stimulus was shown, the red outline flashed twice to denote the area of interest. As a reminder, participants could move the cursor to the image area to show the red outline overlay. On the right side, participants moved the cursor along the rating scale to adjust the rating and click to confirm and proceed to the next trial. Gerard ter Borch (II), *Gallant Conversation (Known as “The Paternal Admonition”)*, 1654. Downloaded from the online repository of the Rijksmuseum, Amsterdam. Cropped to the same framing as the engraving reproduction.

Each participant would first read the instructions and the consent form before the actual experiment. Then they would perform 10 practice trials to get familiar with both the interface and the variety of stimuli. Their task was to rate one of the five attributes regarding the selection marked by a red outline (see Figure 3). Each participant just rated one attribute (e.g. softness) in one condition (e.g. original), in two different media (engraving and painting). The order of trials was randomised across participants.

The interface was designed to minimise the influence of the red outlines: they would first flash twice when the trial started. Then the participant could receive a reminder by moving the cursor to the image. When participants moved the cursor to the right side, the red outlines disappeared and the cursor controlled the rating scale automatically. They could click to rate and proceed to the next trial. Clicking on the image was disabled to avoid accidental ratings.

### Data Analysis

We first performed validity checks for the raw data. We excluded participants who spent <1s on average for each trial. This threshold was based on previous experience (van Zuijlen et al., 2020) in our group. It is very likely that too short answering time means clicking without paying attention, which can result in noisy data. After filtering, each session had on average of 24.4 participants. Then we performed z -score normalisation on the rating data per participant, so that we could later combine data from different participants with different internal scales, and reduce noise. For further analysis, we always used the mean score across all the participants for each material selection.

## Results

**Figure 4.**
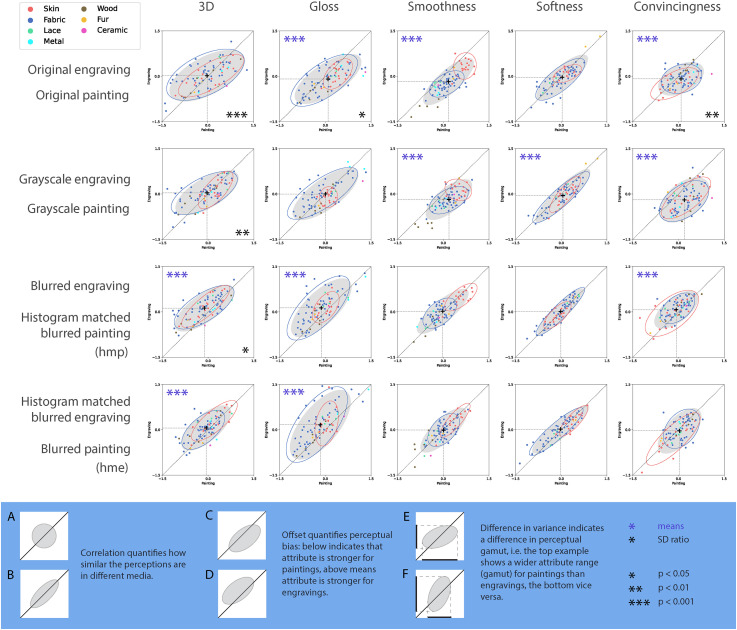
Results overview. Each subplot is an experimental session. Row one to row four represent original, greyscale and two histogram-matched conditions, respectively. Each data point in these scatter plots represents the mean rating of engravings as a function of the mean rating of oil paintings of the same material selection. Each data point is colour-coded with respect to a material category, as indicated in the legend on the top left corner. The ellipses are confidence ellipses from bivariate normal distributions kept constant at 1.96 standard deviation. The grey ellipses are based on all data, the blue and red ellipses denote fabric and skin, the two largest material categories. The legend on the bottom with the blue background illustrates a few possible scenarios. Purple asterisks on the top left corner in a given subplot indicate that the mean ratings for oil paintings and engravings were significantly different for that session. Black asterisks on the bottom right corner of a given subplot indicate that the standard deviations for oil paintings and engravings were significantly different from each other for that session.

The overall results are summarised in Figure 4. Each subplot presents the results of one experimental session with each data point denoting the mean ratings ( z -score) of a given material selection. The x -coordinates denote painting ratings, and the y -coordinates denote engraving ratings. These scatter plots allow for various qualitative inferences that can be made by the eye, but do require some prior intuitions that we will try to provide before discussing the data in more detail. A visual explanation is also given at the bottom of Figure 4.

The scattered data are summarised by covariance ellipses. The grey ellipse denotes all data and the red and blue ellipse denote the subsets of skin and fabric, respectively. The position of the ellipse with respect to the diagonal denotes a perceptual bias: A point above the diagonal line implies that engravings were rated higher than oil paintings and vice versa. An example where this is robustly present is the smoothness data in the original condition: almost all data points are clearly below the diagonal indicating that participants judged materials in paintings to be smoother than in engravings.

A second characteristic, besides position denoting the perceptual bias, is the correlation itself. For example, it can easily be seen that the correlation between painting and engraving is higher for softness than for convincingness. High correlations suggest that the individual ratings judgements are preserved across medium change: something soft in a painting is also perceived as soft in the engraving, which is less so for convincingness. Therefore, this correlation points to a perceptual constancy with respect to the medium.

A third quality of the ellipses is the slope, which indicates whether the range of judgements is different for the two media. If the slope is smaller than one, the perceptual range in the paintings is larger than that for the engravings, which can for example be observed for three-dimensionality judgements in the original (top) condition.

In the following section, we will statistically verify the qualitative observations that we just made. The section after that is devoted to differences between the materials skin and fabric.

### Oil Paintings Versus Engravings

Looking at the overall data in Figure 4, it can be seen that the major axis of the fitted ellipses always points in the positive direction. This is in line with the finding that all correlations are positive and significant ( p<.001 ) ranging from 0.45 to 0.90 (with a mean of 0.71). The correlation coefficients are shown in Table 3.

**Table 3.** Bivariate probability density functions statistics.

	3D			Gloss			Smoothness			Softness			Convincingness		
	Mean	SD	Corr	Mean	SD	Corr	Mean	SD	Corr	Mean	SD	Corr	Mean	SD	Corr
ori_P	− 0.02	0.61	0.59	0.09	0.54	0.69	0.17	0.41	0.79	0.03	0.42	0.81	0.08	0.37	0.45
ori_E	0.02	0.42		− 0.09	0.41		− 0.17	0.39		− 0.03	0.37		− 0.08	0.27	
bw_P	− 0.04	0.52	0.66	0.00	0.54	0.73	0.18	0.38	0.70	0.05	0.45	0.89	0.20	0.41	0.49
bw_E	0.04	0.37		0.00	0.43		− 0.18	0.33		− 0.05	0.44		− 0.20	0.37	
hmp_P	− 0.13	0.48	0.72	− 0.13	0.49	0.71	− 0.02	0.42	0.81	− 0.02	0.38	0.90	− 0.07	0.36	0.64
hmp_E	0.13	0.38		0.13	0.48		0.02	0.37		0.02	0.38		0.07	0.31	
hme_P	− 0.06	0.40	0.69	− 0.17	0.50	0.66	0.01	0.38	0.76	− 0.02	0.47	0.90	0.04	0.36	0.63
hme_E	0.06	0.37		0.17	0.57		− 0.01	0.37		0.02	0.40		− 0.04	0.37	

All correlations have p -value <.001.

P indicates oil painting and E indicates engravings.

The significance of the t tests is indicated by the purple stars in Figure 4.

The ratio of the standard deviation of engravings and that of oil painting varies between 0.69 and 1.14 (with a mean of 0.71). The ratio is smaller than 1 for 17 out of 20 ratios, suggesting that in most cases, the standard deviation for the oil paintings is larger than that for engravings. Levene’s test shows that only five ratios are significant with ratios varying between 0.69 and 0.79: original 3D ( p<.001 ), original gloss ( p<.05 ), original convincingness ( p<.01 ), greyscale 3D ( p<.01 ) and hmp 3D ( p<.05 ). In the above significant cases, oil paintings have a broader range of perceived attributes than engravings. These sessions are marked with black asterisks on the bottom right corner of the corresponding plots in Figure 4. Note that there is a tendency for this ratio to increase towards one from the first row (original condition) to the last row (hme). This is particularly visible in the 3D column.

The means of the oil painting and engraving ratings determine the centroid of the ellipses. In Figure 4, the black plus signs indicate the position of the centroids of grey ellipses (all data). The corresponding values can be found in Table 3. To test for significance, we performed 20 paired t tests for unequal variances. To compensate for the increased chance of Type I error from multiple t tests, we applied Bonferroni correction, and set the critical α value at 0.05/20=0.0025 . For the original condition (the first row in Figure 4), there was no significant difference between paintings and engravings for three-dimensionality and softness. However, oil paintings were rated significantly higher for glossiness, smoothness and convincingness (all with p<.001 ).

**Figure 5.**
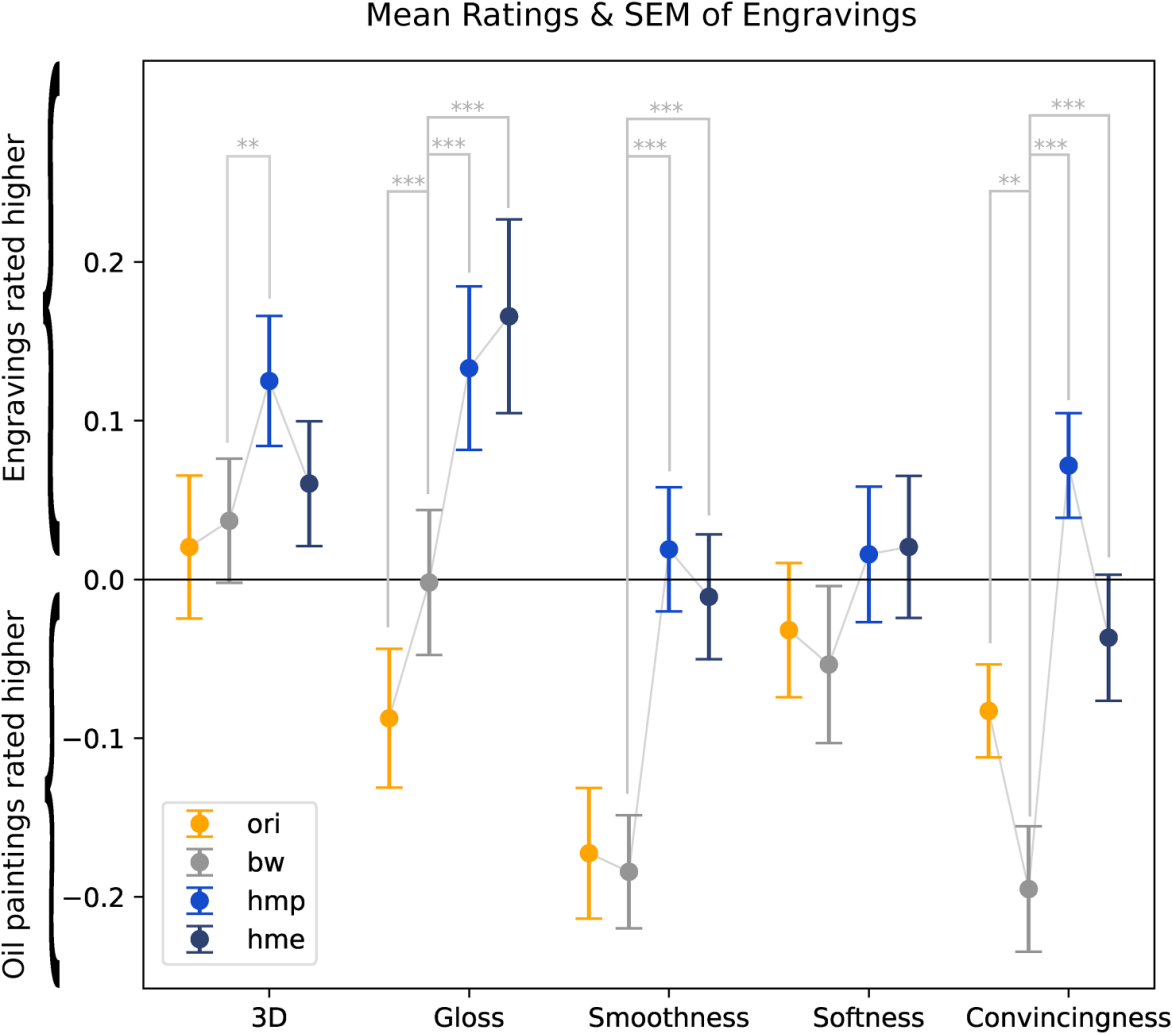
Mean ratings and standard error of means of engravings. It can be seen as a streamlined visualisation of Figure 4, showing the overall trend for the engravings. Positive values indicate engravings were rated higher, negative values indicate oil paintings were rated higher. Since we used z -score data, the means of oil paintings were always equal to the negative means of engravings, as shown in Table 3, hence we only plotted engravings. The lines for oil paintings and engravings would be symmetrical about the x -axis. The light grey asterisk signs indicate the significance of differences between conditions: ** p<.01 ; *** p<.001 . For clarity, only the significance between original and greyscale is indicated, as well as the significance between greyscale and the two histogram-matched ones (hmp and hme).

In Figure 5, the mean ratings are shown for all conditions, which essentially presents the streamlined information of Figure 4, with less distraction from other statistical properties. On the y -axis only the engraving ratings are shown as the painting ratings are the opposite due to the z -transformation. It can thus be viewed as a relative difference. Even more in this representation, it can be seen that gloss, smoothness and convincingness are all judged significantly higher in paintings than in engravings.

After removing the colours resulting in the greyscale condition (second row in Figure 4, grey data in Figure 5), there was no significant change from the original condition for three out of five attributes: three-dimensionality, smoothness and softness. For glossiness, engravings were rated significantly higher after removing colours; for convincingness, engravings were rated significantly lower, both are marked with light grey asterisk signs in Figure 5.

When we applied blurring and luminance histogram matching, the differences between paintings and engravings changed rather substantially. Three-dimensionality was larger for engravings than paintings, while in the original and greyscale versions there was no significant difference between the two media. Glossiness was also larger for engravings than paintings while the reverse was true for the original condition. The differences in smoothness vanished, which also holds for softness although in the original condition there already was no difference. Lastly, the convincingness was significantly higher for engravings than paintings in one condition (hmp), and non-significant in the other histogram-matched condition (hme), while in the original and greyscale condition the paintings were judged as more convincing.

### Comparison Between Material Categories

To investigate possible differences between materials, we compared the results for fabric and skin. These materials were best represented with 54 and 18 elements, respectively. We mainly focus on qualitative observations about the bivariate normal distributions, denoted in Figure 4 by the red and blue confidence ellipses for skin and fabric, respectively. Looking at the red (skin) and blue (fabric) ellipsoids, we observe various configurations: overlapping (some position and size), enclosing (one smaller and within the area of other) or complementary (inhibiting different areas). Note that these three possibilities also hold for a uni-dimensional representation of the data, that is, on one of the axes. The first row of Figure 4 shows the data for the “original” condition and illustrates the three qualitative configurations well: three-dimensionality and (to a lesser extent) convincingness show overlapping data, glossiness and softness show encapsulating configurations and smoothness shows a complementary configuration with the ellipse for skin systematically above that for fabric. The interpretation is relatively straightforward and will be presented in the Discussion section.

## Discussion

As Figure 4 and Table 3 show, all conditions and attributes show positive correlations, indicating that oil painting and engraving media elicit similar perceptions for these five attributes. In other words, engravers did an excellent job of replicating the oil paintings and provoking similar perceptions for the five attributes we tested, although engraving is a challenging medium with only monochromatic lines and dots. This finding is in line with the conclusions from Delanoy et al. (2021) and van Zuijlen et al. (2020) that different media provoked similar material perception. This “perceptual constancy over medium” for both our study and van Zuijlen et al. (2020) could be partially driven by semantic information, as Fleming et al. (2013) has shown: relatively similar perceptual spaces were found for mere material classes defined by their word as by their photographic representations. Yet, the role of semantics vanishes when trying to explain the variance within material categories, such as fabric or skin.

The differences between oil paintings and engravings are mainly found in their means and standard deviations. Different means indicate different levels of perceived attributes. Different standard deviations indicate different perceived ranges of certain attributes. In the original condition, the oil paintings always show a broader range of perceived attributes, regardless of the significance of variance differences. To be precise, in almost all sessions (17 out of 20) oil paintings have a broader perceptual gamut than engravings. Bousseau et al. (2013) found that the range of perceived gloss in painterly renderings is narrower than that in realistic renderings. Our current study shows that the perceived ranges of three-dimensionality, gloss and convincingness in engravings are significantly narrower than those in oil paint. For the original pictures, three out of five attributes showed a smaller range for engravings, but after removing chromatic information (colour), only three-dimensionality showed this difference between painting and engraving, and differences vanished completely in one of the two histogram-matched conditions. It should furthermore be noted that, although not significant, for Gloss, the perceptual range of engravings seems to trump that of paintings in the case of histogram matching.

### Comparisons in the Original Condition

The first row in Figure 4 shows the comparison between oil paintings and engravings for the original condition. For glossiness, smoothness and convincingness, representations in oil paintings were rated significantly higher, meaning materials in oil paintings were perceived as glossier, smoother and more convincing. The difference in convincingness is to be expected: the combination of colourlessness and the visibility of hatching lines likely lacks the convincingness found in oil paintings. Less expected is that convincingness showed a larger perceptual range in paintings. This finding is less straightforward to explain than the larger perceptual range for three-dimensionality and gloss (discussed in more detail in the next paragraphs). As gloss and three-dimensionality vary in reality, it makes sense to depict these variations and the painting medium apparently affords the depiction of a larger variety of pictorial attributes than engraving. However, convincingness is not an attribute of a pictorial object but rather an overall quality of the depiction itself. Convincingness does not vary in reality, as reality itself is an ultimate aim achieved through convincingness. There does not seem a need or motivation for a larger convincingness range in paintings than in engravings. Therefore, this range difference may reflect that differences in style may be larger within paintings than engravings, which would be an interesting observation. This would imply that in copying a painting into an engraving, idiosyncratic style elements are lost and depictions converge towards a more homogeneous “engraving style.” It seems feasible to investigate this conjecture empirically, although it is beyond the scope of the current study.

**Figure 6.**
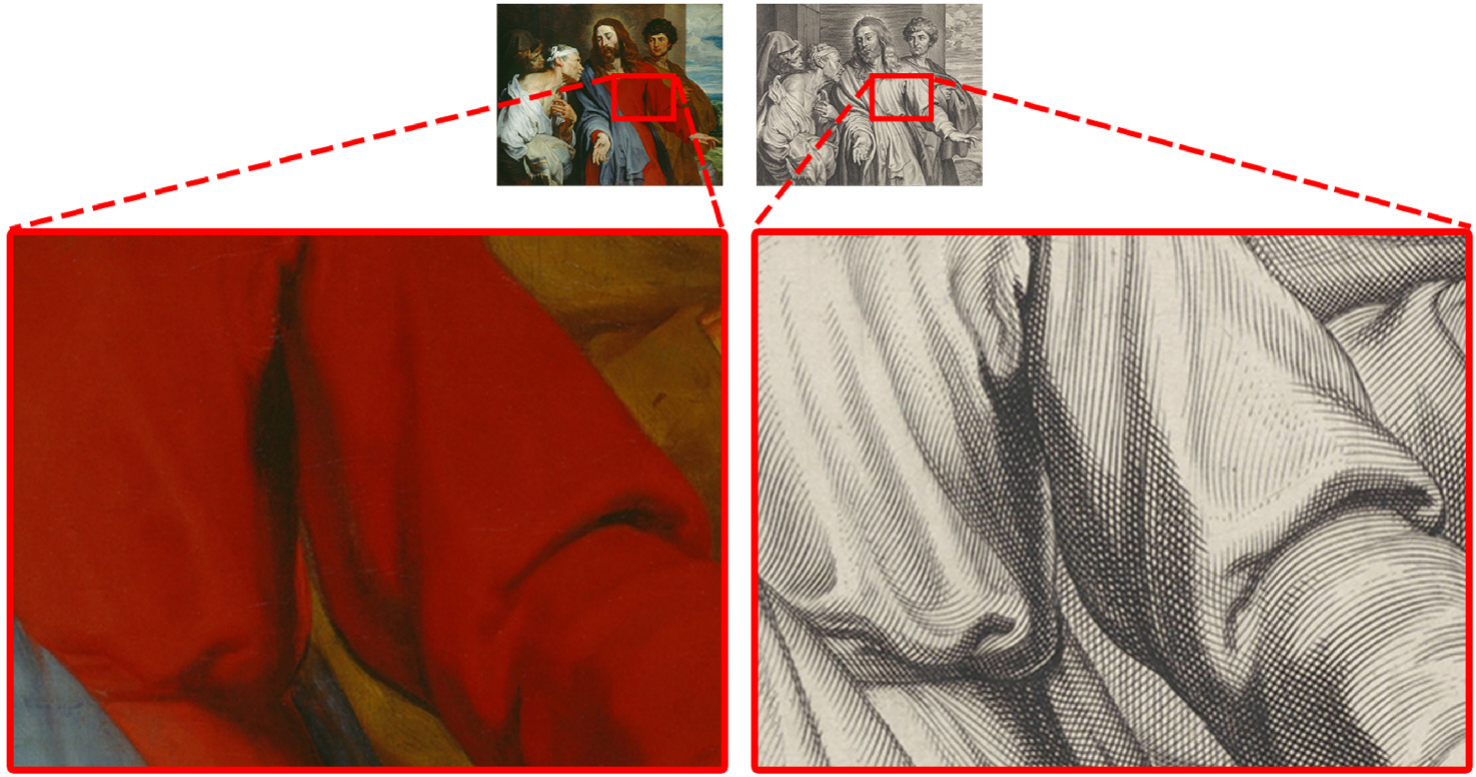
A zoomed-in look at details. The engraving on the right shows visible engraving lines. Oil painting on the left: Anthony van Dyck, *Christ healing the paralytic*, 1619. Engraving on the right: Pieter de Jode (II), *Christ healing the paralytic*, 1628–1670.

The difference in mean ratings for smoothness is rather large. One possible explanation is that in the original condition, the brushstrokes in oil paintings were fine and not very visible, while engravings have visible engraving lines (see an example in Figure 6). As discussed in the introduction, we previously found an interaction between the smoothness of the medium (visible brushstrokes) and pictorial smoothness (of the motif) in a study on apple depictions (Zhao et al., 2023). Although we specifically instructed the participants to rate the smoothness of the depicted material, it could be a similar case of observers unable to discount the smoothness of the medium while judging the smoothness of the motif.

**Figure 7.**
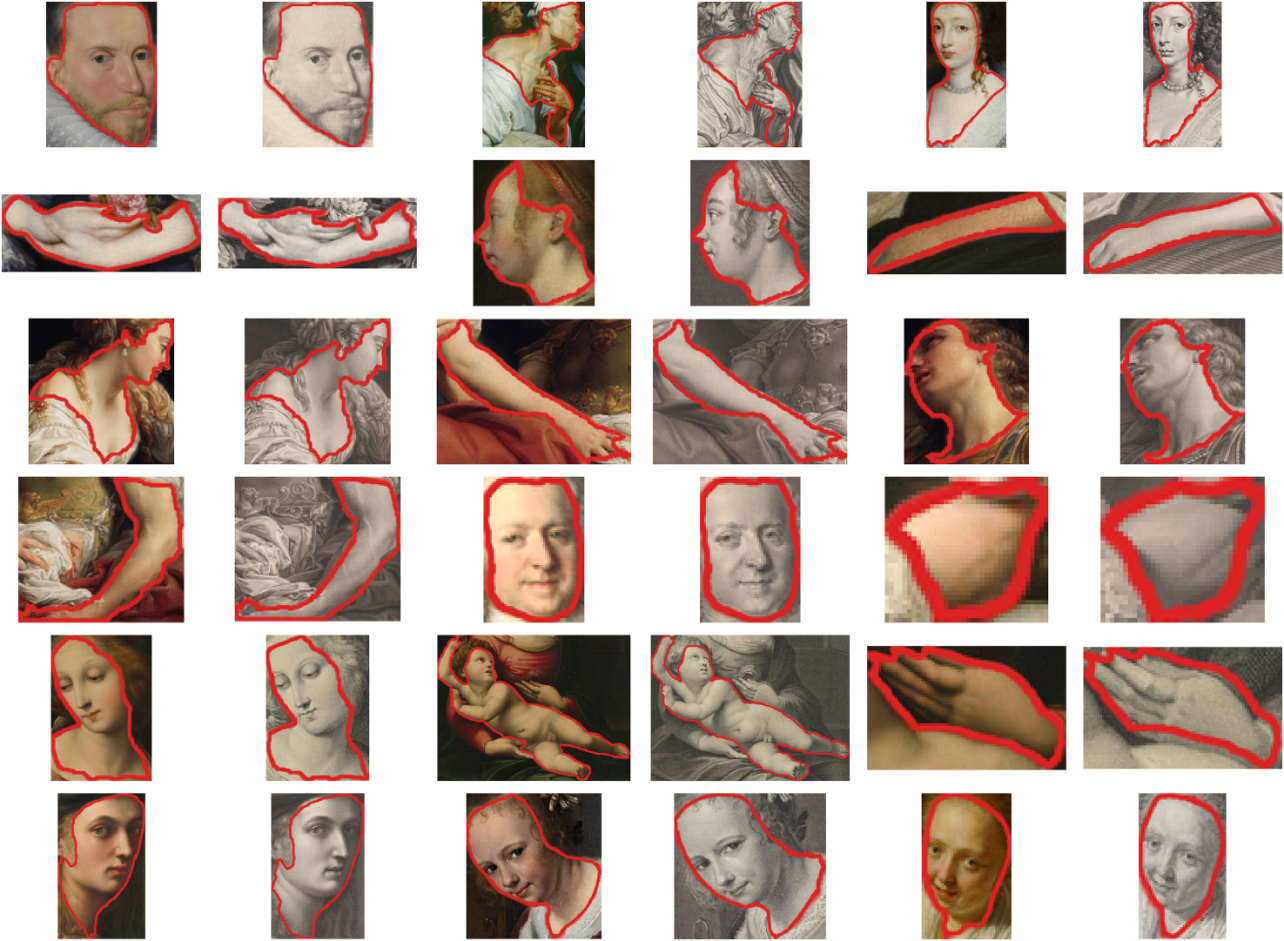
Examples of skin fragments from paintings (on the left) and engravings (on the right).

The difference in perceived gloss is more challenging to explain. Indeed, the painter possesses more control over the gloss parameters, especially being able to vary the amount of blur at the edge of highlights. That would not explain an overall higher gloss rating for paintings, but it could contribute to the larger perceptual range as found by comparing the variances (indicated by the black asterisk in Figure 4). If we observe the material-specific categories (blue ellipse for fabric and red ellipse for skin), we observe that skin dominates the gloss bias. Apparently, painted skin appears more glossy than engraved skin. A look at the skin fragments in Figure 7 may suggest a possible explanation. While both engraving and painting make use of tonal differences to articulate shape and material, it seems easier to disentangle the specular reflections from the shading patterns in paintings than in the engravings.

**Figure 8.**
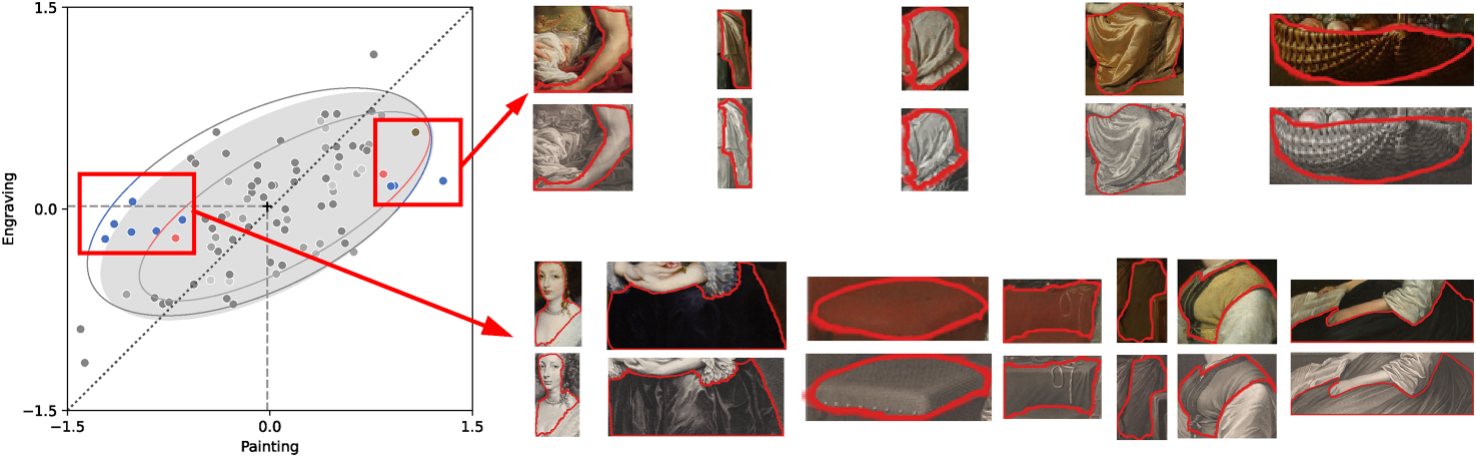
Some examples from the three-dimensionality ratings in the “original” condition illustrate a potential cause for the difference in perceptual range between paintings and engravings. From the left rectangle: some objects in paintings with low three-dimensionality ratings hardly show tonal contrast while their engraved equivalents do. From the right rectangle: some objects that show similar levels of shading and detail.

As for three-dimensionality and softness, we did not find differences in mean ratings between paintings and engravings. However, we did find a larger perceptual range for three-dimensionality in paintings. To understand the three-dimensionality range difference we show some stimuli that seem responsible for this effect in Figure 8. The stimuli that elicited low three-dimensionality ratings for paintings in comparison to engravings (left rectangle in the figure) all seem to show objects that were painted without contrast, rather homogeneous without much shading detail in comparison to their engraved counterparts. The stimuli in the right rectangle should show the opposite effect, that is, very 3D in paintings and less so for engravings. Indeed, the paintings show well-articulated shading patterns, especially in comparison to the paintings with low three-dimensionality. However, the engravings for this second group of stimuli look quite similar to the paintings; they also show shading articulation. If anything, the paintings seem to include both shading and (cast) shadowing while engravings seem mostly involved with shading patterns. In sum, when looking at individual stimuli we can indeed see a relatively large range in three-dimensionality for paintings and a much shallower range (more similar) for engravings.

### Effect of Colour Removal and Histogram Equalisation

We manipulated the images to reduce the two most prominent differences between paintings and engravings: chromatic information and the luminance histogram. The colour manipulation was performed for the obvious reason that engravings lack colour information. The rationale behind the luminance histogram equalisation was to reduce the difference in global luminance statistics (for the whole image) such as mean luminance, contrast (as quantified by the variance) and skewness.

By only removing colours, the evoked perceptions of the two media did not change much compared to the original condition (the second and first rows in Figure 4, or the grey and orange points in Figure 5). This suggests that colour did not affect perception much. It was the blurring and histogram equalisation that had a more substantial overall impact. We will now discuss the results in more detail.

A somewhat surprising result is that the difference between paintings and engravings in convincingness was enhanced instead of mitigated when removing chromatic information. Many facets can underlie the perception of convincingness. In the computer science literature, the closest equivalent to “convincingness” is “realism” and Rademacher et al. (2001) found that shadow sharpness and surface texture visibility contribute significantly towards the perception of realism, both in photos and renderings. Although there is interesting literature comparing realism across various art styles, Hagen (1986) mainly focuses on the depiction of pictorial space and various types of perspective. An extension towards computer rendering (Ferwerda, 2003) offers three varieties of realism: physical, photo(metric) and functional realism. While broadening the scope towards other formal elements than pictorial space, the categorisation seems too coarse to offer an explanation for our finding. One plausible speculation could be that in the original condition, the styles are so far apart that each is judged on its own merit but as differences become smaller, the two media are more directly compared by the observers. Again, this is mere speculation in need of further empirical evidence. What is certain is that when we removed differences in luminance histograms, convincingness differences vanished and for half of the data even reversed: when histograms were matched to the painting the engravings were judged to be more convincing. Initially, this manipulation aimed at histogram matching to equalise the luminance characteristics, such as mean, variance (i.e., contrast), and skewness. However, a side effect was the necessity to blur the engravings in order to compute a continuous histogram. In hindsight, the blurring alone would have merited an independent manipulation as in the case of convincingness the effect may well have depended on the visibility of engraved lines.

For three-dimensionality, the colour removal did not cause much difference: the larger perceptual range persists for paintings and the mean three-dimensionality ratings are again not significantly different between paintings and engravings. However, when applying the luminance histogram equalisation, we see that the perceptual range difference vanishes for half of the data (the histograms matched the engravings). This could potentially be due to contrast equalisation. As we showed in Figure 8, this seemed a potential difference between paintings and engravings. Moreover, we found a significant difference in mean three-dimensionality ratings. Given that chromatic information and the (global) luminance distributions are similar between the paintings and engravings, these rather robust findings are likely due to local contrast, that is, the detailed shading contrast on certain objects seems to be stronger in engravings than in paintings.

**Figure 9.**
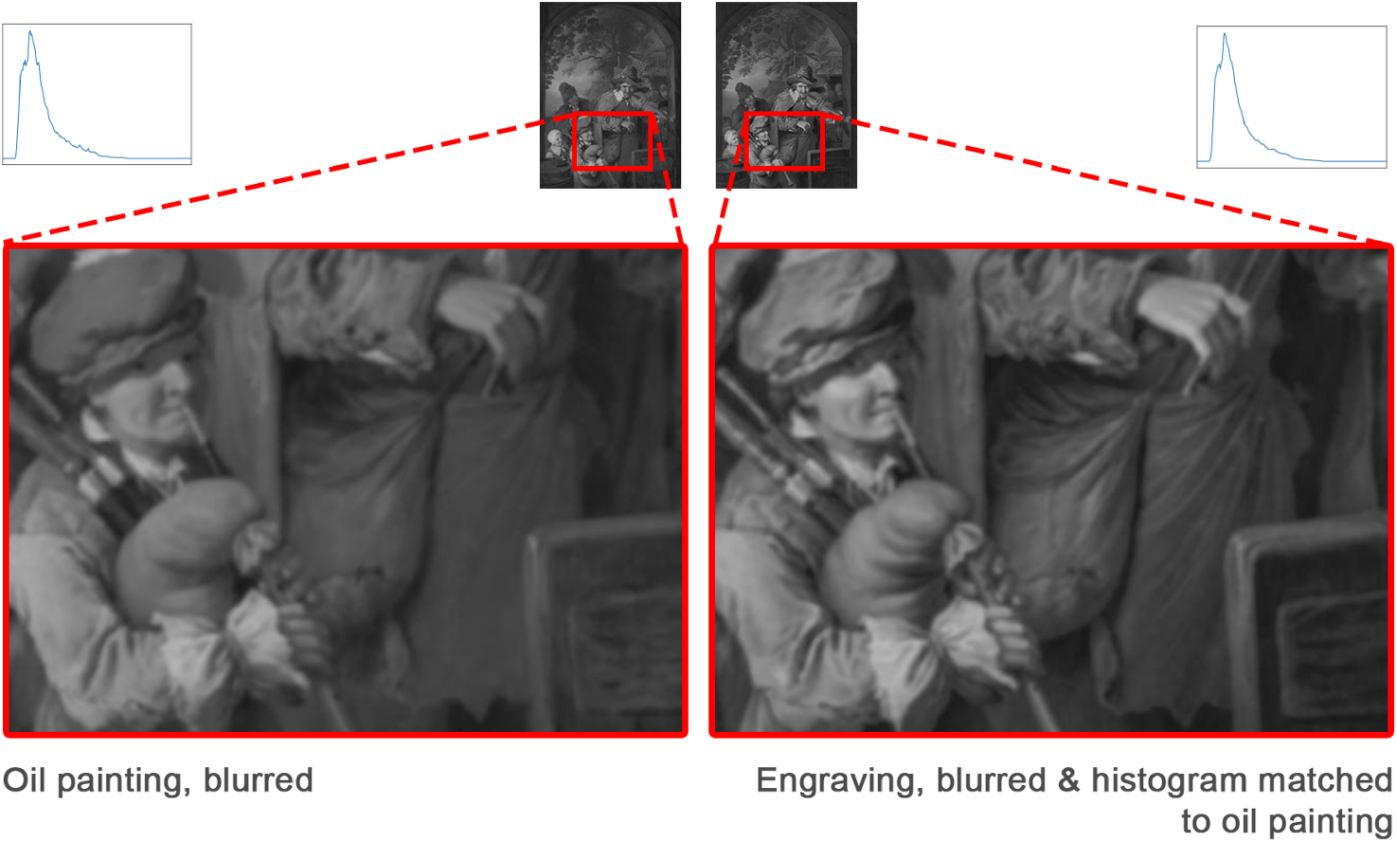
A zoomed-in look at details on blurred oil painting and blurred engraving with histogram matched to oil painting. The engraving on the right shows higher local contrast than oil paintings, although they share the same histogram. Oil painting on the left: Christian Wilhelm Ernst Dietrich, *The Wandering Musicians*, 1745, from The National Gallery, London. Engraving on the right: Johann Georg Wille, *The Wandering Musicians*, 1764, from Rijksmuseum, Amsterdam.

A similar shift in mean ratings was found for gloss perception. While in the original condition, glossiness ratings were higher in paintings than in engravings, removing colour caused this difference to vanish and luminance histogram equalisation even reversed the effect: engravings are perceived to be more glossy. In the original condition, we conjectured that the bias towards paintings could be attributed to the skin, as illustrated in Figure 7. The removal of colour did not seem to change much about the position of the red ellipse (denoting the skin samples) with respect to the diagonal although the position itself shifted downwards. Yet, the vanishing of the mean gloss difference in the greyscale condition seems to be due to fabric samples (engravings show higher gloss) counterbalancing the skin samples (paintings show higher gloss). With the removal of luminance histogram differences the engravings robustly received higher ratings. We believe that this bias is also due to local contrast, as shown in Figure 9. The effect seems similar to the three-dimensionality data, although the underlying mechanism differs: for gloss, the contrast between highlight and background is an important cue (Marlow Anderson, 2013) while for three-dimensionality contrast in general likely plays a role. While a change, in contrast, can theoretically be attributed to either a change in light direction or to a change in depth (Belhumeur et al., 1999), it has been shown that participants often attribute it to shape: Ho et al. (2006) tested surface roughness with a rather coarse texture stimulus and found that increasing contrast by lowering the light direction was attributed to the roughness, that is, depth variation as the texture was rather coarse.

For smoothness, in both original and greyscale conditions, oil paintings received higher ratings. After blurring and histogram matching, the performance of these two media became very similar. One possible explanation is that in both original and greyscale conditions, the visible lines led to less perceived smoothness for engravings. After blurring, the engraving lines became invisible, resulting in similar smoothness ratings between these two media. As mentioned earlier, in a previous study about style perception (Zhao et al., 2023), we found a potential transfer between the smoothness of depicted apple skin and brushstroke coarseness of the medium. Although we cannot dissociate whether smoothness perception similarity relies on blurring or histogram equalisation, we hypothesise that it is indeed due to the vanishing engraving lines. This would imply that again we found a transfer of smoothness/roughness from medium to depicted objects/materials.

Softness was the only attribute in the original condition that neither showed a significant difference between the means nor the variances of paintings and engravings. This changed when we removed colour information: objects were perceived as softer in paintings than in engravings. It is tempting to believe that smoothness and softness are correlated and that the solution of the softness bias towards paintings finds its origin in the smoothness discussion from the previous paragraph. Yet it can be readily inferred that smoothness and softness are judged differently by observers: for smoothness the skin and fabric samples are clearly segregated while for softness there is much overlap. This leaves us with the open question of why painted objects are perceived as softer than engraved objects when colour is removed. The second manipulation (histogram equalisation) let the softness bias disappear again, which could either mean that global contrast or hatching visibility contributed to the bias we found in the achromatic condition. What is furthermore interesting to note is that for softness the correlations were all rather high: in the original condition about 0.8 and in all manipulated conditions about 0.9, as can be read in Table 3 and also observed in Figure 4. These values are all substantially higher than for the other attributes. This implies that the softness of materials is the most medium-invariant attribute.

### Comparison Between Material Categories

As we showed in Section , the two material categories of skin and fabric have different configurations. For three-dimensionality and convincingness, they have an overlapping configuration, indicating similar perceptual ranges. For gloss and softness, they show an enclosing configuration. Skin has a lower glossiness and softness range than fabric. A possible explanation is that fabric is a more diverse material category than skin. It can vary from matte cotton to glossy satin, or from heavy stiff damask to soft silk. Skin, on the other hand, is much more consistent. For smoothness, skin has overall higher values than fabric. The possible explanation is that skin is in general smooth, while fabric is in general less smooth than skin, and can vary in terms of smoothness.

Additionally, for each attribute, the configurations of these two material categories demonstrate similar trends across the manipulations. This suggests fabric and skin have similarly been affected by the colour and luminance manipulations.

### Conclusion

We investigated the perceptual influence of media by measuring judgements about materials, shape and pictorial quality (convincingness). We choose to compare engravings and paintings as they are both famous art media and because of the engraved copies of paintings we could study a similar pictorial scene differently depicted in the respective media. Furthermore, paintings and engravings span an important historical style axis as defined by Heinrich Wölfling who in his “Principles of Art History” (Wölfflin, 1922/2012) defined the first dimension of style and form that between “linear” and “painterly.”

Our overarching interest is how engravers handled the limited boundary conditions of their medium. How to cope with the lack of colour and the binary nature of tonal variations? Indeed, when directly compared to paintings, engravings lack convincingness. But this difference vanishes when the boundary conditions are equalised for the media. Moreover, gloss and three-dimensionality judgements are higher for engravings than for paintings in equalised conditions, and for softness and smoothness perceptual differences vanish. We have hypothesised that engravers show a stronger local articulation of the shading details, which likely compensated, or was meant as compensation, for the lack of colour and smooth transitions afforded by oil paint. A more detailed study on what types of pictorial ingredients engravers use to convey material properties would be highly desirable. Our study has generated a number of other interesting follow-up questions. First, we found more evidence for the interaction between medium and motif, in our case for smoothness perception. Second, as three-dimensionality relies on both shading and shadowing, the clear visibility of these two is necessary for an optimal three-dimensionality percept. For engravings, however, the discernibility between shading and shadowing seems to be limited. Thirdly, a difference in the depiction of skin became apparent where there again seemed to be dissociation difficulties for engravings, this time between shading and highlight. Fourthly, although this may be more art-historically interesting: what is the role of paint degradation when comparing engravings and paintings, particularly the local shading patterns? It seemed that some parts of the paintings were rather dully shaded while their engraved counterparts were highly articulated. Was this the engraving compensating as just discussed, or was the original painting equally articulated? A future study could investigate whether some of our paintings did in fact degrade over time, although this may require some technical art history effort.

In conclusion, engravings can render materials and shapes well and elicit similar perceptions as oil paintings. Nevertheless, there were some differences in performance for portraying certain attributes, as well as differences in perceptual range, which has resulted in interesting new research leads. In addition, we showed the role of colour and luminance distribution via manipulations of colour removal, blurring and histogram equalisation. The manipulations close the gap between them. In some cases, engravings even show advantages over oil paintings.

## Supplemental Material

sj-pdf-1-ipe-10.1177_20416695241261140 - Supplemental material for Material perception across different media—comparing perceived attributes in oil paintings and engravings Supplemental material, sj-pdf-1-ipe-10.1177_20416695241261140 for Material perception across different media—comparing perceived attributes in oil paintings and engravings by Yuguang Zhao, Jeroen Stumpel, Huib de Ridder and Maarten W. A. Wijntjes in i-Perception
