# Supplementary figures and images for: Material perception across different media—comparing perceived attributes in oil paintings and engravings
Source: Iperception. 2024 Aug 3;15(4):20416695241261140. doi: 10.1177/20416695241261140 (PMC11297522; doi:10.1177/20416695241261140)

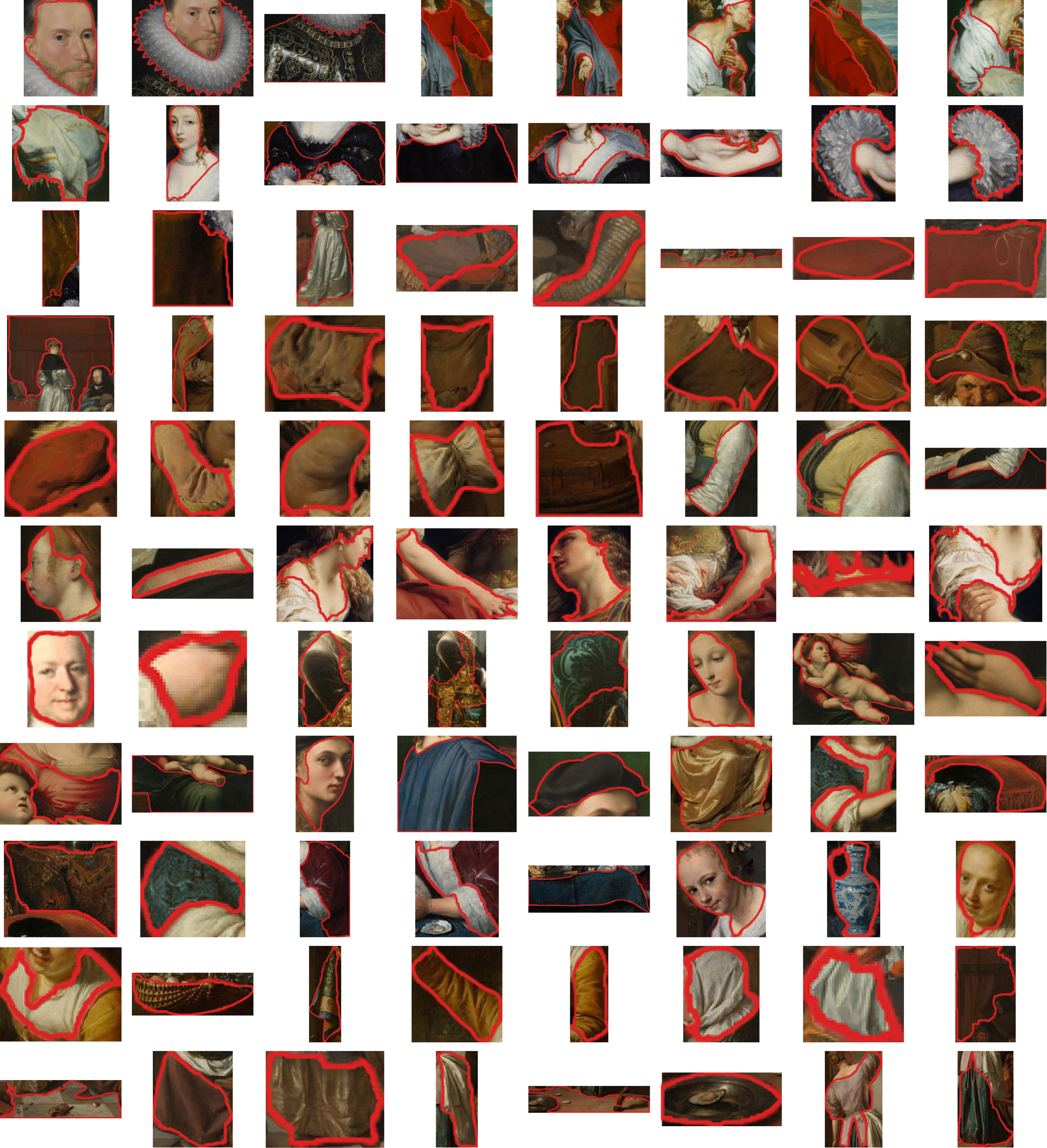

Supplement: sj-pdf-1-ipe-10.1177_20416695241261140 - Supplemental material for Material perception across different media—comparing perceived attributes in oil paintings and engravings [file sj-pdf-1-ipe-10.1177_20416695241261140.pdf]
